# The Potential of Soluble Human Leukocyte Antigen Molecules for Early Cancer Detection and Therapeutic Vaccine Design

**DOI:** 10.3390/vaccines8040775

**Published:** 2020-12-18

**Authors:** Amy L. Kessler, Marco J. Bruno, Sonja I. Buschow

**Affiliations:** Department of Gastroenterology and Hepatology, Erasmus MC, University Medical Center Rotterdam, 3015 GD Rotterdam, The Netherlands; a.kessler@erasmusmc.nl (A.L.K.); m.bruno@erasmusmc.nl (M.J.B.)

**Keywords:** soluble human leukocyte antigens (sHLA), biomarker, immunopeptidomics, liquid biopsy, mass spectrometry, cancer immunotherapy

## Abstract

Human leukocyte antigen (HLA) molecules are essential for anti-tumor immunity, as they display tumor-derived peptides to drive tumor eradication by cytotoxic T lymphocytes. HLA molecules are primarily studied as peptide-loaded complexes on cell membranes (mHLA) and much less attention is given to their secretion as soluble HLA–peptide complexes (sHLA) into bodily fluids. Yet sHLA levels are altered in various pathologies including cancer, and are thus of high interest as biomarkers. Disconcordance in results across studies, however, hampers interpretation and generalization of the relationship between sHLA levels and cancer presence, thereby impairing its use as a biomarker. Furthermore, the question remains to what extent sHLA complexes exert immunomodulatory effects and whether shifts in sHLA levels contribute to disease or are only a consequence of disease. sHLA complexes can also bear tumor-derived peptides and recent advancements in mass spectrometry now permit closer sHLA peptide cargo analysis. sHLA peptide cargo may represent a “liquid biopsy” that could facilitate the use of sHLA for cancer diagnosis and target identification for therapeutic vaccination. This review aims to outline the contradictory and unexplored aspects of sHLA and to provide direction on how the full potential of sHLA as a quantitative and qualitative biomarker can be exploited.

## 1. Introduction

The field of cancer immunotherapy has undergone major advances in recent decades, facilitated by innovative strategies such as immune checkpoint inhibition, therapeutic vaccines, oncolytic viruses and adoptive T cell therapy. The success rates of cancer therapies, however, rely heavily on their window of opportunity, which is often at the early stages of malignancy [1,2,3]. Easy and rapid detection methods are therefore needed that can accurately diagnose patients at an early stage. Ideally, biomarkers to detect cancer are both highly sensitive and specific for the disease. For many biomarkers, high specificity is difficult to ensure because general inflammatory processes may confound biomarker use. Furthermore, biomarkers should be minimally invasive to prevent unnecessary risk and physical impact.

Human leukocyte antigen (HLA) molecules, also known as major histocompatibility complex (MHC) molecules, are essential for effective immune responses. Their function is to display tumorigenic or pathogenic peptides on the cell surface membrane to flag cells for recognition and killing by cytotoxic T lymphocytes (CTLs). HLA class I (HLA-I) molecules are expressed by almost all nucleated cells, whereas HLA class II (HLA-II) molecules are generally only expressed by specialized cells called antigen-presenting cells (APCs). HLA-I molecules present peptides derived from endogenously synthetized proteins, whereas HLA-II molecules present those derived from exogenous proteins taken up by the APC. However, the process of cross presentation, limited to APCs, enables the presentation of exogenous peptides on class I molecules as well.

Aside from canonical expression as HLA–peptide complexes on cell membranes (mHLA), they can also be secreted by cells in a soluble form (sHLA). Since the discovery of sHLA molecules in 1970 [4], it became apparent that sHLA levels are often altered during disease and various studies ensued to evaluate their diagnostic potential in cancer. A general trend of elevated sHLA levels exists in cancer patients. However, the reports diverge, especially when it comes to their correlation with progression and prognosis. These varying results have up until now made it difficult to understand the relationship between sHLA and cancer. The mechanisms behind sHLA induction and release are still not fully understood and neither is their immunological function. Obtaining answers to these questions would increase the applicability of sHLA as diagnostic tool. Additionally, it is known that sHLA molecules, like mHLA molecules, carry peptides in their binding groove. These peptides, when derived from tumor-specific proteins, might provide important clues for tumor detection, but can also support target identification for antigen-specific immunotherapies.

This review aims to bring attention to sHLA complexes as a potential approach for early cancer detection and for the development and/or personalization of cancer therapy. First, the diagnostic value of circulatory sHLA will be discussed together with the pitfalls that currently hamper its translation into the clinic as a biomarker. Next, the knowledge so far on mechanisms behind sHLA release and sHLA-mediated effects on immunity will be reviewed. Lastly, how recent advances in sHLA peptidome analysis can contribute to the development of therapeutic cancer vaccines will be described.

## 2. sHLA in Malignancies

With a few exceptions, a general trend of elevated sHLA levels in cancer patients is observed across a variety of cancer types. Here, we will review studies so far that document the presence of these sHLA forms in bodily fluids in cancer. Studies on the different HLA types (i.e., classical HLA-I (-A, -B and -C), non-classical HLA-I (-E,-F and -G), and HLA-II (DR)) will be discussed individually as their tissue expression and hence the mechanism underlying their secretion is likely different. The focus will mostly be on classical HLA-I as it is best known for its polymorphic peptide carrying abilities and it is expressed on nearly all cells of the body. Other sHLA types will also be briefly touched upon for which either significantly fewer studies have been performed (i.e., HLA-E, HLA-F and HLA-II) or, in case of HLA-G, for which the literature is vast and comprehensively reviewed elsewhere [5,6,7]. For the sake of clarity, solid tumors and hematological malignancies are also discussed separately.

### 2.1. Classical sHLA Class I

Classical HLA-I complexes (i.e., HLA-A, -B, -C) drive the cellular immune response and can in theory be secreted by all nucleated cells that express HLA-A, -B and -C, albeit at greatly variable levels. Non-immune cells in peripheral tissues express relatively low levels, whereas immune cells, especially professional APCs, express high levels [8]. Studies that investigate classical sHLA-I complexes almost exclusively use the pan-HLA-I W6/32 antibody, which also recognizes HLA-E, -F and –G complexes [9,10,11,12]. Levels of non-classical HLA, however, tend to be much lower and thus the contribution of non-classical sHLA-I complexes to total sHLA-I amounts is limited when studied combined. For this reason, despite that the focus here is on classical sHLA-I complexes, we refer to them as sHLA-I in total.

Thus far, only a limited number of studies has been performed on classical sHLA-I in solid tumors. In bodily fluids of patients suffering from lung and gastrointestinal cancers as well as uveal melanoma, sHLA-I was elevated compared to healthy individuals (Table 1, upper part). Levels in some cases also had prognostic value. In one study, sHLA-I levels in bodily fluids from patients with various malignancies, however, were not significantly increased from those with infections or other pathologies including liver cirrhosis, cardiac or renal failure or autoimmunity, although a trend was seen [13]. This study indicates that inclusion of a control group with non-malignant disease is important to determine whether elevated sHLA-I levels are cancer specific or rather by-products of for example inflammatory processes. Cancer specificity of elevated sHLA-I is therefore in most studies unproven due to the lack of such control groups. Further of interest is that atypically, in gastric cancer sHLA-I levels were lower than in healthy subjects [14], without an apparent explanation.

Slightly more data have been collected on classical sHLA-I complexes in hematological cancers (Table 1, lower part). It is reasonable to assume that hematological tumor-derived sHLA complexes are more efficiently released into the patient’s circulation than sHLA from solid tumors, which are often surrounded by a stromal environment. Besides, white blood cells, given their immunological function, may express relatively high levels of classical HLA-I and hence be more prone to secrete it. Indeed, all studies on classical sHLA-I in hematological malignancies unambiguously reported elevated sHLA-I levels compared to healthy controls, and some also found higher levels in relation to more advanced disease. Interestingly, association of sHLA-I with survival/clinical behavior can differ between clinically related cancers. A significant association of sHLA-I levels with survival was found in Non-Hodgkin’s lymphoma (NHL), but not in Hodgkin’s disease (HD) [15], and sHLA-I levels significantly correlated to clinical behavior in acute myeloid leukemia (AML) but not advanced myelodysplastic syndrome (MDS) [16]. This could be due to the distinct immune processes underlying each disease, but this needs to be further clarified. Of note, also for these studies assessing sHLA in hematological cancers, no other non-malignant diseases have been included as controls, making it difficult to determine whether observations were cancer specific.

### 2.2. Non-Classical sHLA Class I

Non-classical sHLA-I complexes have mostly been investigated in solid tumors rather than hematological cancers. This is likely because of the fact that these complexes have an immune-suppressive function and harbor a selective expression pattern. In homeostatic conditions, few mature tissues express HLA-G: the cornea [25], the thymic medulla [26], and pancreatic islets [27]. HLA-F expression normally seems to be limited to trophoblasts and activated lymphocytes [28,29]. Surface HLA-E expression relies on the presence of HLA-I leader sequence-derived peptides, suggesting that all nucleated cells may express HLA-E at their surface at variable levels [30]. All three non-classical HLA-I complexes, however, have been demonstrated to have increased expression in tumor tissues [31].

As already indicated, reports on sHLA-G in cancer up until 2017 have been extensively reviewed elsewhere [5,6,7]. To complete the overview of sHLA-G in cancer, in Table 2 all studies were included that were either performed after 2017 or that were missing in the referenced papers. In summary, most recent studies assessing sHLA-G in the context of solid tumors found increased levels in patient samples [5,6,7,32,33,34] (Table 2, upper part). A few studies also included non-malignant diseases as their control cohorts [35,36,37,38,39]. In all but two studies, sHLA-G levels were significantly higher in cancer patients compared to the non-malignant controls. In one of these cases, however, sHLA-G levels in benign colorectal diseases (i.e., hyperplastic polyps, adenomas and IBD) significantly differed from healthy controls as well, indicating that for HLA-G non-malignant disease controls are also highly important [35].

Interestingly, sHLA-G levels followed an opposite pattern in thyroid cancer patients. Patients were less often serum sHLA-G positive than healthy controls [40], and those that suffer from invasive growth secreted significantly less sHLA-G than those that do not [41]. This is in contrast with increased surface HLA-G expression on thyroid cancer tissue [42]. This may be explained by differential induction of surface and soluble HLA-G, or by environmental factors that affect the access of sHLA-G to the blood, such as stromal density and vascularity. For instance, in colorectal cancer (CRC) patients, vascular invasion was associated with sHLA-G levels above a certain threshold [43]. To our knowledge, although likely, the association between stromal density and blood sHLA levels has not yet been investigated. In addition to thyroid cancer, plasma sHLA-G levels were also lower in breast cancer patients. However, these were non-treatment-naïve samples, thus the relationship between sHLA-G and breast cancer is unclear [44].

In a few studies, sHLA-G levels also positively correlated with more advanced disease stage [5,6,7,33,38,45,46]. In CRC, however, sHLA-G levels were found to have contrasting prognostic values depending on the TNM stage [43], illustrating that using HLA-G as a solid tumor biomarker may not always be straightforward.

In hematological malignancies, sHLA-G levels have been found elevated compared to healthy controls and occasionally also related to poorer disease course (Table 2, lower part). Notably, elevation of sHLA-G may occur after chemotherapy [47]. 

Lastly, sHLA-E and sHLA-F have only been the topic of five and two studies, respectively, equally covering solid and hematological cancers (Table 3). Similar to the other sHLA types, sHLA-E and -F are mostly elevated in cancer patients. In contrast to classical sHLA-I and sHLA-G, however, higher sHLA-E and sHLA-F levels were in one study associated with better survival rates [48]. How and whether this relates to their specialized functions are unknown. Yet thus far, no non-malignant controls were taken along in any non-classical sHLA-E and -F studies, making it difficult to conclude their true value as a biomarker in hematological cancers. 

### 2.3. sHLA Class II

The information on sHLA-II complexes in cancer is scarce (Table 4). There are two studies on sHLA-DR with contrasting results, one in melanoma and one in acute lymphoblastic leukemia (ALL). As ALL is a B-cell derived cancer, the elevated sHLA-DR levels in this disease may be explained by a high expression of HLA-DR on cancerous pre-B cells which could have been the source of sHLA-DR. Based on these studies, it is currently not possible to conclude the value of sHLA-II molecules for early cancer detection.

### 2.4. Confounding Factors Affecting sHLA Levels and/or Detection

The observed relationships of sHLA and cancer presence or prognosis may be largely attributable to the biology of the different sHLA molecules and/or may derive from malignancy-specific factors. However, several discrepancies between studies were observed that are rather difficult to explain from a mechanistic point of view. We believe that a lack of standardization across sHLA studies can, at least in part, explain varying results and may thus hamper studies attempting to use sHLA as a biomarker or trying to explain mechanisms of sHLA secretion of action (discussed below).

**Figure 1:**
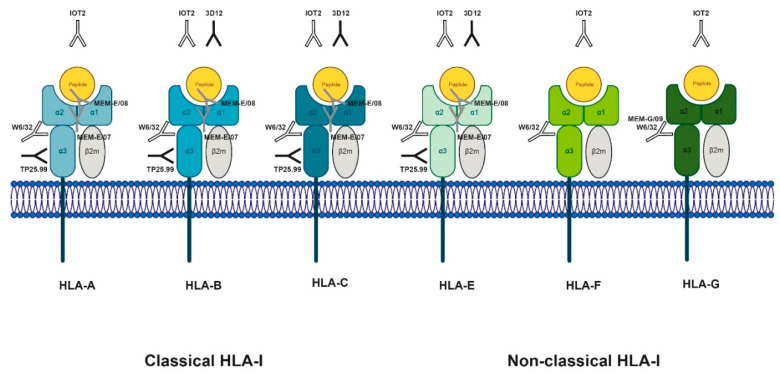
**Reported target specificity of anti-HLA-I antibodies frequently used in sHLA studies**. The binding domain of each antibody is represented by its location relative to the HLA complex. The color of the antibody indicates its specificity for native (conformational) or linear (non-conformational) epitopes. Open = native, grey filled = linear, and black filled = both. Studies reporting the depicted antibody specificities are mentioned in the main text. Antibodies for which no specific epitopes were reported are depicted above the HLA complex.

Firstly, the lack of standardized detection methods greatly obstructs comparison of sHLA studies. One of the concerns in the field of sHLA research is antibody specificity. The most commonly used pan-HLA-I antibody W6/32 is regularly described as specific for native HLA-A, -B, -C and –G molecules [62], despite numerous reports that W6/32 also recognizes native HLA-E and -F molecules (Figure 1) [9,10,11,12]. Because sHLA-G and sHLA-E/-F are usually found at a concentration of 1 to several orders of magnitude lower than sHLA-ABC (Table 1), results from this antibody will mostly reflect sHLA-ABC. Yet, HLA types may be differentially regulated across disease types and stages and thus understanding the specificity of the applied antibody(s) will be crucial to optimally exploit the diagnostic value of sHLA in cancer. For example, Contini and colleagues employed antibodies W6/32 and TP25.99 to measure sHLA-I molecules by ELISA in AML patients [24]. While W6/32 is a pan-HLA-I antibody, TP25.99 is not (Figure 1) [63,64]. This setup thus overlooked sHLA-G which was later demonstrated to be elevated in AML patients [54]. During the first international workshop on sHLA antigens, a fairly good correlation between sHLA-I assays from different laboratories was found despite the use of different antibodies and/or protocols [65]. However, the absolute values of sHLA-I measured in identical samples did differ among the laboratories, underlining the necessity of a better international protein standard. Additionally, the specificities of the anti-HLA-E MEM-E and 3D12 antibodies have also been subject to discussion, as some evidence was presented that these antibodies recognize more than one HLA type [9,11,66], making interpretation of results difficult (Figure 1). Unfortunately, no epitope information on IOT2 could be recovered, impairing interpretation of its binding specificity.

For the classical sHLA-I molecules, the efforts to develop quantification methods have been extensive [65,67,68], yet a reliable commercial kit is not readily available. Most ELISA formats used in sHLA-I studies have been developed by the academic researchers themselves and pre-dominantly use the W6/32 antibody in combination with either another class I-reactive antibody, or an anti-β2m antibody (Table 1). For sHLA-G molecules, efforts have been made to standardize detection methods. In 2004, a wet workshop was organized to validate two ELISA formats that could detect either HLA-G5 alone, or HLA-G5 + sHLA-G1 [69]. Antibody pairs 5A6G7 + W6/32 and MEM-G/09 + anti-β2m were verified as reliable reagents to measure HLA-G5 or HLA-G5 + sHLA-G1 respectively in a reproducible manner. Most studies investigating sHLA-G levels seem to dominantly use the antibody pair MEM-G/09 + anti-β2m, or a commercial ELISA kit designed to recognize sHLA-G1 and HLA-G5 (Table 1). It should be noted, however, that this kit allows sHLA-G quantification in units/mL rather than ng/mL, and that a commercial sHLA-G standard is not available yet [70]. Class II molecules are arguably the least studied form of sHLA molecules, and especially in context of malignancy their value is rarely investigated. Hassan and colleagues argue that this may be a consequence of the lack of commercial sHLA-DR kits [61], even though there have been efforts to create a quantitative assay for sHLA-II molecules [71]. Taken together, the development of well-defined and reliable ELISA kits that are commercially available would greatly facilitate sHLA research.

Secondly, the used sample type and its subsequent processing can majorly impact measurements of low abundance proteins such as sHLA. Plasma and serum differ in concentration of certain proteins; probably due to the tendency of some proteins to aggregate and get caught in clots during serum preparation [72]. Indeed, sHLA-G levels are higher in EDTA-plasma samples compared to paired serum and heparin-plasma samples, while the latter contain nearly equal amounts. Although sHLA levels in paired serum and CPDA-1 plasma samples correlated strongly, these differences in absolute amounts do warrant caution for the selection of blood collection tubes and anti-coagulants [65,70,73]. Furthermore, a delay time in blood processing as short as 4 h after blood collection may influence protein levels, as has been shown for certain cytokines in both serum and plasma [74]. Lastly, freezing and thawing specimens may also influence sample quality. A comparison of plasma and serum samples before and after around-the-world shipping on dry ice suggested that plasma specimens had a higher stability of sHLA-I levels than serum specimens [65].

Different cancer treatment regimens may also influence sHLA levels in patients. These therapy-induced fluctuations may overshadow the true cancer-driven alterations in sHLA quantities, and hamper the investigation into its potential as a biomarker. Examples are systemic interferon (IFN)-α treatment that increased sHLA-G serum and sHLA-I plasma levels in melanoma and chronic myeloid leukemia (CML), respectively [52,75]. In these cases sHLA-G and sHLA-I levels returned to normal after 10 months or 2–4 months of ongoing treatment respectively. Although IFNs are known to upregulate mHLA-I expression levels [76,77,78,79], it remains unclear how exactly IFNs increased sHLA release in these studies and why it decreased after months of therapy. Suggested was that sHLA may have been released upon IFN-mediated cell death or that IFNs more specifically induce sHLA secretion via upregulation and shedding of mHLA or through alternative splicing [52,75]. No explanation was provided for the drop of sHLA-I after prolonged treatment. Possible IFN treatment resistance mechanisms hampering IFN receptor signaling may have been involved [80]. In addition to IFNs, another example of treatment effect was a rise in sHLA-I observed after the initiation of chemotherapy in a cohort of acute myeloid leukemia (AML) patients [24]. In AML, elevations in sHLA-I quantities were most pronounced in the first 5 days and returned back to pre-chemotherapy levels after 9 days. In contrast to this, no effect of cytostatic treatment on sHLA was found in melanoma, while in NHL and HD patients sHLA-I serum levels rather decreased upon initiation of chemotherapy or radiotherapy [20,52]. Taken together, past or ongoing treatment regimens should always be reported and corrected for during investigation of sHLA as diagnostic marker.

Lastly, the HLA allotype distribution in a cohort can also affect sHLA differences. For example, serum sHLA-I levels have been found elevated in HLA-A*24+ individuals compared to other allotypes [14,81]. Of note, a broad range of studies report on no significant differences in sHLA levels between different age groups and gender [13,19,35,36,37,52,56,60,61].

### 2.5. Future Diagnostic Utilization of sHLA

The lack of standardization and the relatively low number of studies for each sHLA type and malignancy impedes immediate translation of sHLA to the clinic as a diagnostic tool. Ideally, multiple studies that investigate the same cancer type in different cohorts should arrive at the same conclusion by using the same ELISA formats. Moreover, the inclusion of a non-malignant control cohort is essential to determine the cancer specificity of these complexes and has been lacking in a substantial number of studies. Extending upon this, the number of studies that perform a receiver operating characteristic (ROC) analysis (plotting the relation between sensitivity and specificity) to assess the discriminative power of sHLA as a biomarker is limited. Although only partially informative due to the lack of proper non-malignant control groups, the described area under the curve (AUC) for sHLA-G to discriminate cancer patients from healthy controls was significantly different from chance (AUC = 0.5) in all reporting studies and ranged from 0.6 to 0.842 [33,35,36,39,44,50]. From these studies, three report on a good discriminative power of sHLA-G between malignancy and other, non-malignant diseases, although two of those mixed non-malignant control samples with healthy controls for ROC analysis [35,36,39]. For sHLA-I in total, only 1 study quantitatively assessed its discriminative power and observed a sensitivity and specificity of 88.2% and 85.5%, respectively [17]. In future research, ROC analyses should be performed with the proper control cohorts to better assess the true discriminative power of sHLA.

Nonetheless, evidence for the potential use of classical sHLA-I as an early diagnostic tool has been substantiated albeit thus far most convincingly in hematological tumors; perhaps due its easy access to the circulation. sHLA-G might be more suitable to detect solid tumors due to its cancer-restricted expression pattern and seemingly enhanced discriminative power over non-malignant disease. Whether sHLA potentially can be utilized as a marker for cancer progression or response to therapy needs to be investigated more extensively. To now explore the full potential of sHLA, large-scale monitoring of sHLA levels in the clinic is needed to study their diagnostic value in bigger patient cohorts, possibly in combination with current golden standard cancer biomarkers [32,35].

## 3. sHLA Complexes: Mechanism of Secretion and Immunological Effects

Since their discovery, many have tried to find explanations for the altered sHLA levels in malignancies and their association with disease progression and prognosis. In addition to malignancies, altered sHLA levels have been observed in chronic disease [82], autoimmunity [83,84], and viral infections [85,86]; strongly suggesting that there are shared inflammatory pathways that regulate sHLA. Knowing how sHLA-I and sHLA-II release is regulated and to what extent these complexes affect immune responses is crucial to determine whether their increased presence is merely a consequence of ongoing immunity or whether they also partake in pathophysiological processes.

### 3.1. sHLA-I Complexes

**Figure 2:**
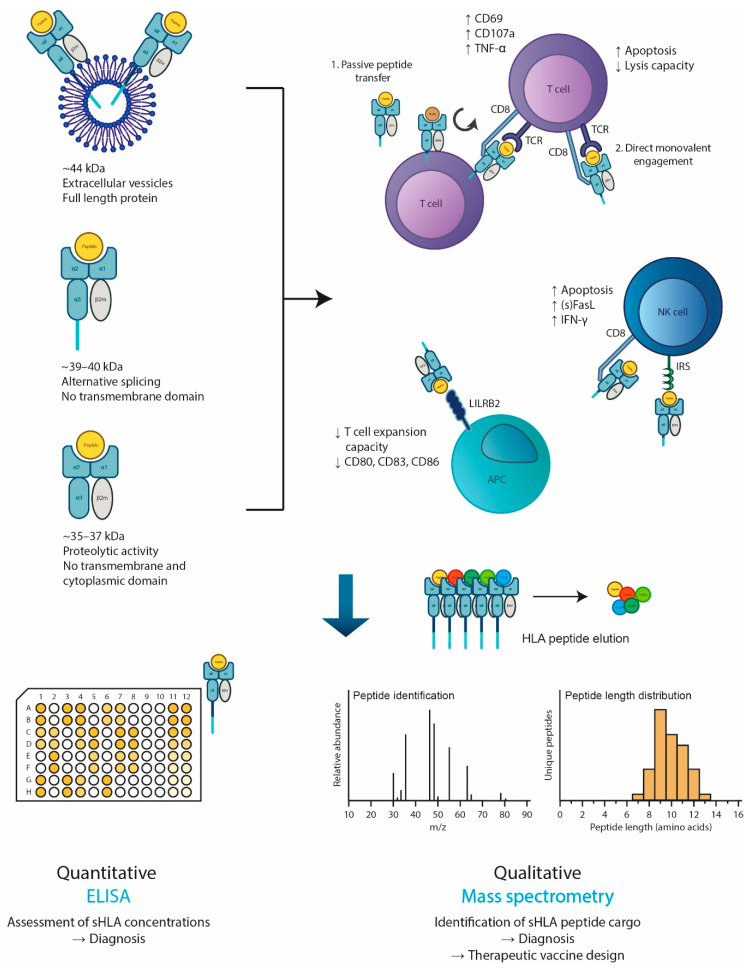
**sHLA-I complexes: mechanisms of release, immunological effects and analysis**. sHLA-I complexes can be secreted into bodily fluids in three molecular forms depending on the mechanism of release. Once released, sHLA-I complexes may encounter T cells, NK cells or APCs and affect their functioning through interaction with their respective receptors. For T cells, two mechanisms of activation have been postulated: passive peptide transfer or direct monovalent engagement. It is currently unknown whether each sHLA-I molecular form can affect immune cells in a similar manner. Quantitative analysis of sHLA complexes is performed (mostly) by sandwich ELISA methods (chapter 2). Qualitative analysis, entailing the identification of peptides and their length, is performed by advanced mass spectrometry as described in chapter 4. See the main text for references to the studies providing evidence for the depicted concepts.

sHLA-I complexes structurally resemble mHLA-I complexes: they are composed of a ~35–44 kDa heavy chain (discussed below) that is commonly associated with a 12 kDa β2-microglobulin (β2m) and a peptide of 8–11 amino acids in length [87,88,89]. There have also been reports of peptide-loaded, β2m-free soluble heavy chains that are able to re-associate with free-floating β2m [90]. sHLA-I heavy chains can have three different molecular weights: 44, 39–40, and 35–37 kDa (Figure 2) [91,92]. The heaviest molecular form represents the intact HLA molecule how it is usually present on the cell membrane of nucleated cells. This form contains a transmembrane domain and is released from cells in a membranous form, likely via extracellular vesicles that are derived from endosomal intraluminal vesicles (i.e., exosomes) or that were formed by budding from the plasma membrane [92,93]. The intermediate form of 39–40 kDa is a splice variant of the full protein that lacks the transmembrane domain [94]. Lastly, the smallest sHLA-I form is generated by proteolytic activity at the cell membrane, resulting in a free-floating extracellular domain [90]. Of note, proteolytic shedding or vesicle-mediated secretion could represent a mechanism for tumor immune escape by reducing cellular mHLA. In contrast, other (epi)genetic or indirect mechanisms by which tumor cells may downregulate mHLA may also abrogate sHLA release and/or alter its peptide content [95].

Because the secretory mechanisms for the three molecular forms are different, it is likely that they are differently affected and regulated by inflammation, although common patterns may also exist. For example, IFN-γ stimulation has been found to increase both metalloproteinase-dependent cleavage of sHLA-I complexes [96] and alternative splicing leading to sHLA-I release [97]. In Sjögren’s syndrome, however, sera of patients with inactive disease contained none to low levels of the 39 kDa and 44 kDa isoforms, in contrast with sera from those with active disease [83]. Although this requires further investigation, this implies that the diagnostic value of sHLA could be improved by studying each form separately rather than all forms together.

Although a one-on-one relationship between mHLA protein levels and sHLA secretion could be expected, this is not necessarily the case. Cellular mHLA and sHLA levels do not always correlate [50], and in lymphocytes sHLA-I secretion was dependent on *de novo* RNA and protein synthesis, suggesting that the increase in sHLA-I is not merely due to the release of *a priori* synthesized mHLA-I complexes [98]. Tumor cells, whilst known to downregulate surface HLA expression, can secrete sHLA complexes in either a spontaneous fashion or upon stimulation with pro-inflammatory cytokines [91,99,100,101,102]. B and T lymphocytes can also secrete sHLA complexes in vivo and in vitro but only under antigenic and mitogenic conditions [98]. In this respect, not all cytokines that induce mHLA protein expression are also capable of stimulating sHLA secretion by cancer cells [99]. For example, while both type I IFNs and IFN-γ upregulated mHLA-I expression, only IFN-γ induced sHLA-I secretion, suggesting that upregulation of mHLA-I does not by default lead to more sHLA-I secretion but rather depends on independent active release mechanisms. Taken together, sHLA-I upregulation in disease and different cell types may be regulated by multiple pathways and does not solely rely on expression of surface HLA-I complexes.

sHLA-I complexes have been shown to exert immunological effects on various cell types, including T cells, NK cells and APCs (Figure 2). Firstly, CD8+ T cells can be activated by all monomeric, peptide-loaded sHLA-I complexes (i.e., classical and non-classical), characterized by higher T cell expression levels of CD69, CD107a, and TNF-α [103,104]. The notion that sHLA-I in a monomeric form can still activate T cells despite the absence of T cell receptor (TCR) cross linking can be explained by the finding that during sHLA-I-T cell interaction, sHLA-I-bound peptides are efficiently transferred onto T cell surface-resident cognate HLA-I molecules which then mediate T cell activation via the TCR (Figure 2) [103,104]. Peptide transfer from sHLA-I to T cell mHLA-I did not require the internalization of the peptide-loaded sHLA-I complex and was impaired by the addition of exogenous competing peptides [103]. Intriguingly, the efficiency and kinetics of sHLA-I-mediated T cell activation via peptide transfer was close to that of free peptides, suggesting a highly efficient mechanism that calls for further investigation.

Contrasting these studies on T cell activation by sHLA are those reporting inhibitory effects. Multiple groups have found that engagement of sHLA-I complexes with T cells rather diminished T cell lysis capacity and/or triggered T cell apoptosis (Figure 2) [62,105]. The CD8/Fas/FasL pathway plays a crucial role in the latter [24,62]. On their own, sHLA-I, sHLA-A2 and sHLA-G1 molecules have nearly equal potency to induce T cell apoptosis and IFN-γ production by NK cells [62]. Yet sHLA-G1 is likely only a small contributor to apoptosis as its serum levels are relatively low.

Secondly, sHLA-I can also engage with NK cells through the CD8 receptor and activate them in a dose-dependent manner, first resulting in IFN-γ production (but not cytotoxic activity) and ultimately NK cell apoptosis (Figure 2) [62,106,107]. Further, with regard to IFN-γ stimulation and apoptosis of NK cells, sHLA-G1 is equally efficient as total sHLA-I [62,106,107]. Notably, sHLA-I-mediated effects can be simultaneously inhibited by ligation of co-expressed members of the inhibitory receptor superfamily (IRS) [107]. These IRS bind in an allele-specific manner to sHLA-I complexes; thus the balance between activating and inhibiting signals may depend on the composition of sHLA and the expression levels of IRS members [105].

Lastly, sHLA-I can also affect the functioning of APCs such as monocytes and monocyte-derived dendritic cells (moDCs), presumably via the Leukocyte Immunoglobulin-Like Receptor (LILR) family (Figure 2) [85]. Upon sHLA-I exposure, moDCs have reduced capacity to expand allogeneic T cells and express less co-stimulatory molecules on their cell surface.

### 3.2. sHLA-II Complexes

sHLA-II (and murine sMHC-II) complexes, like their membrane counterparts, consist of an alpha and beta chain that form a heterodimer of approximately 60–65 kDa [108,109] and bear peptides of ~15 amino acid in length [109,110]. sHLA-II molecules have also been reported as 43 kDa and 18 kDa isoforms, the former of which may represent a non- or de-glycosylated variant [109,111]. In one study, murine sMHC-II complexes were highly stable, as multiple efforts to dissociate the complexes were without success [109]. Whether this was an exception or is true for sHLA-II complexes in general is unknown.

Like sHLA-I, sHLA-II can be found in serum [108]. One study demonstrated no obvious link between sHLA-II allotypes and levels of release [111], but this has not been addressed in sufficiently large cohorts. sHLA-II complexes, in addition to serum, can consistently be found in sweat, tears and saliva of healthy donors, whereas sHLA-I cannot or at low levels only [83,111,112]. It is therefore likely that the mechanisms behind sHLA-I secretion differ from those responsible for the secretion of sHLA-II. Furthermore, as sweat, tears and saliva generally do not contain HLA-II-bearing immune cells, it is remarkable that sHLA-II complexes are constitutively present in these bodily fluids. How sHLA-II ends up in sweat, tears and saliva is unclear. The general mechanism behind sHLA-II release, however, has also not yet been elucidated. As far as we know, no HLA-II alternative splicing has been demonstrated suggesting that sHLA-II complexes are predominantly derived from mHLA-II complexes, possibly from exosomes or other extracellular vesicles [93]. In vitro APCs indeed secrete exosomes bearing class II complexes [113,114], however, the contribution of exosomes to sHLA levels in circulation has been challenged [115].

Consistent with the restricted expression of HLA-II by antigen-presenting cells and activated T cells, sHLA-II complexes are likely released by activated CD4+ T cells, DCs and B cells [108,113], which can be further enhanced by macrophages or cognate DC-T cell interaction [113]. Trophoblast cells can also release sMHC-II complexes upon stimulation with IFN-γ, indicating that sHLA-II can also be derived from cell types that do not overtly express mHLA-II [116].

The immunological role of sHLA-II is not as extensively investigated as of sHLA-I, and most of the studies have been performed in murine models. In one study it was found that sIA^d^ molecules can increase proliferation of macrophages, B cells and T cells and decrease antigen-specific IgM production upon stimulation with mitogens [117]. Additional stimulation is seemingly crucial for sHLA-II-mediated immune effects, as sHLA-DR molecules in vitro are not able to stimulate T cell clones by themselves, but rely on the presence of an additional stimulant [108]. In another murine model, sMHCII^d^ and sMHCII^k^ molecules also decrease autoantibody production and thereby reverse the effects of broken immune tolerance in vitro and in vivo [118]. In contrast with the first study, however, in this and another model, sMHCII^d^ molecules decreased proliferation and/or apoptosis of antigen-stimulated splenic cells in vitro [109,118,119]. This is further accompanied by an increase in CD4+ CD25+ cells, CD4+ CTLA-4+ cells, and IL-10, and decrease in CD4+ CD28+ cells and IL-2. Taken together, more research, especially in the human setting, is needed to conclude the role of sHLA-II in disease.

## 4. sHLA Peptides for Cancer Detection and Therapeutic Vaccine Design

Similar to mHLA molecules, sHLA molecules can also carry peptides in their binding groove. The collection of peptides found on HLA complexes is referred to as the ‘*HLA peptidome*’ or *‘immunopeptidome’.* These peptides can derive from normal, endogenously synthesized self-proteins tolerated by the immune system, but in case of cancer also from aberrantly expressed or mutated tumor-related proteins. These tumor-related HLA–peptide complexes can flag tumor cells for recognition by T cells and are therefore of particular interest for immunotherapy design. Importantly, tumor cells can release sHLA complexes which may contain information about their tissue of origin in the form of peptides derived from organ-specific and/or tumor-specific (mutant) proteins [88,89,91,110,120]. Therefore, the sHLA peptidome in cancer has potential to serve as a “liquid biopsy” for the non-invasive detection of malignancy and at the same time the identification of targets for therapeutic vaccine design.

Mass spectrometry (MS) is currently the only accurate technique to explore the HLA peptidome at a large scale. In short, peptides are eluted at low pH from immunoprecipitated HLA complexes and analyzed by liquid chromatography tandem mass spectrometry (LC–MS/MS) (Figure 2) (recently extensively reviewed by [121]). By using reference databases, peptide sequences and their source proteins are inferred based on their mass spectra [121]. If desirable, peptide quantification can also be performed.

The isolation and subsequent processing for LC–MS/MS analysis has been proven feasible and reproducible for sHLA derived from cell culture supernatants, plasma, and serum samples; yielding up to 2000–2500 peptides from 2.5 to 3 mL of plasma or 9 mL of whole blood [88,89,91,110,120]. Furthermore, sHLA-I complexes are relatively stable in circulation [91]. Owing to their low prevalence in blood, the yield of sHLA-DR- and sHLA-DQ-derived peptides is significantly lower; ranging between 34 and 180 identified peptide sequences from 2.5 to 3 mL plasma [110]. This amount further decreases after the removal of contaminating peptides.

In contrast to the total amount of sHLA (described above), one study reported that the number of recovered sHLA-I peptides did not differ between plasma and serum samples [89]. This might have depended on the type of anti-coagulant used, however, and should be validated in other studies. When working with plasma samples, it is important to control for peptide contamination from blood clotting and plasma proteins. This problem can largely be resolved by adding protease inhibitors to the samples directly after collection and by post MS/MS analysis filtering steps [91]. Conversely, serum sHLA-I complexes contain more peptides derived from proteolytic activities during blood clotting, even after extensive washing [89].

To exploit sHLA peptide cargo for cancer detection and immunotherapy development, it is essential that sHLA complexes derive from the tumor (microenvironment) and that the sHLA peptidome represents the tumor’s mHLA peptidome. Generally, it is to be expected that mHLA-I and sHLA-I peptidomes from the same cells match relatively well, as two out of the three sHLA-I secretion pathways involve the transition of mHLA-I into sHLA-I (i.e., shedding of vesicles and proteolytic cleavage). This could be different for sHLA-I generated by alternative splicing, which lacks a transmembrane domain. Removal of the transmembrane domain of the HLA-A*02:01 molecule, however, did neither significantly alter its maturation kinetics nor the binding motif, post-translational modifications or source protein distribution of bound peptides [89,122], suggesting that the sHLA-I peptidome is not different for alternatively spliced sHLA-I complexes. Similar to mHLA, approximately 90% of recovered peptides from sHLA-I and sHLA-II complexes are around 8–11 and 15 amino acids in length, respectively [88,89,110,120]. Moreover, nearly all sHLA-I-derived peptides fit the binding motifs of the donors’ HLA allotypes, indicating that identified peptides were indeed true sHLA-I-binders [89,91,110,120].

Importantly, in blood cancers, it was found that the sHLA-I peptidome can share up to 86% of the peptides with the mHLA-I peptidome [91]. Although a significant fraction of the sHLA-peptidome consisted of self-peptides [120,123], cancer-specific peptides could be recovered from patient-derived sHLA-I complexes, including tumor-associated antigen (TAA)-derived peptides that may be of interest as antigenic targets for therapeutic vaccination or adoptive T cell therapy [88,91,120,123]. More than 60% of sHLA-I peptides that originated from TAAs were also found on tumor mHLA-I complexes. Additionally, a selection of sHLA-I derived peptides not present in non-malignant plasma samples, decreased by at least twofold upon tumor resection [120]. Despite the fact that this was only true for a minority of the sHLA peptidome, it does indicate an opportunity to monitor tumor presence via sHLA-I peptidome analysis as these peptides were present in sufficient amounts to be sensitively measured.

The combination of organ-specific and tumor-specific sHLA-I-derived peptides may allow for a sensitive detection of tumor presence and its anatomical location. Thus, LC–MS/MS-based sHLA-I peptidome analysis seems a promising quick and non-invasive approach for both the detection of malignancy and for the discovery of therapeutic vaccine targets. For the latter, identified TAA peptides may be used to develop personalized therapies. Alternatively, upon the identification of one TAA-derived peptide in a patient’s sHLA, other, more commonly found peptides derived from the same TAA can be used to compose a more universal or semi-personalized off-the-shelf (mix and match) therapy. Of note, for use as immunotherapeutic targets, TAA-peptides identified on sHLA complexes would require further testing for immunogenicity.

Along the same line, sHLA-II peptides could also be of interest when derived from tumor cells or tumor-resident APCs that may have ingested tumor cells. Tumor immunity largely depends on CD8+ T cell responses and tumor cell surface HLA-I expression as HLA-II is generally not expressed by tumor cells, although exceptions exist [124]. Yet, CD4+ T cells can significantly enhance the CD8+ T cell anti-tumor response by providing ‘help’ signals during T cell priming [125], and thus TAA-derived sHLA-II ligands would be of interest. Current HLA peptidome analysis techniques, however, would need to improve further to facilitate the identification of suitable HLA-II-restricted antigen targets.

Both sHLA-I and sHLA-II peptidome analysis and their use as a biomarker or to identify immunotherapeutic targets greatly benefit from recent improvement of LC–MS/MS sensitivity and also the implementation of quantitative methods [121,126]. Furthermore, efforts to optimize sample processing are consistently made to improve yield and accuracy [121,127]. Collectively, these factors significantly increase sensitivity and thus diminish the need for high-volume patient material, which could now render sHLA peptidome analysis feasible for clinical application.

## 5. Conclusions

The use of sHLA for diagnostic and therapeutic purposes is promising. sHLA levels are consistently found to be elevated in cancer patients, although cancer specificity remains uncertain due to imperfect study design. First, many sHLA studies lack power due to relatively small study cohorts. Secondly, a tumor prognostic marker identified in a discovery cohort should be ideally validated in an independent validation cohort and publications reporting the marker should follow the REporting recommendations for tumor MARKer prognostic studies (REMARK) guidelines [128]. To date, none of the studies currently published validated their findings. Thirdly and perhaps most importantly, the inclusion of appropriate control groups, including those with non-malignant disease, is crucial to determine the value of sHLA as a (prognostic) cancer biomarker. After all, the purpose of such a biomarker is to distinguish early stage malignancy from non-malignant diseases and health, which requires high tumor specificity. In this respect, further specificity may be obtained by studying different forms of sHLA separately.

The lack of method and reagent standardization is currently another major challenge in sHLA research. This is probably the least problematic for sHLA-G, for which a commercial ELISA kit is available. For total sHLA-I and sHLA-II, however, further standardization is needed. Currently, many discrepancies can be attributed to varying protein standards, sample processing protocols and antibodies used. By standardizing these confounding factors, hopefully the true value of sHLA as a prognostic cancer biomarker can be uncovered.

It is evident that sHLA complexes exert immunological effects; the question remains, however, to what extent this affects tumorigenesis. Although sHLA complexes have been shown to activate T cells, it is plausible that the net result of sHLA release is immune inhibitory based on its potent apoptotic potential and ability to hamper cytolytic activities. Yet, the immunological outcome of sHLA release may depend on other immune-regulating factors in the (micro)environment. Like for mHLA, the concomitant presence of inhibitory and co-stimulatory factors (e.g., receptors or cytokines) could tip sHLA-mediated effects towards either activating or inhibitory. Furthermore, high stromal density could hamper widespread sHLA release, resulting in localized effects only, rather than systemic.

Improved sample processing protocols, sensitive machinery, and advanced analysis methods have contributed to the rapid development of immunopeptidomics. One of the biggest benefits of such analyses is that apart from the identification of potential therapeutic targets, sHLA peptide cargo could also aid in cancer diagnosis in addition to the quantitative assessment of sHLA by ELISA and could potentially increase sensitivity and specificity. sHLA-II peptidome LC–MS/MS analysis still has a way to go before it reaches the accuracy and efficiency that is currently reached for sHLA-I. For both, however, the setting of smaller tumors in early stage malignancy will be especially challenging as it is reasonable to assume that the amount of tumor-specific peptides decreases with smaller tumor size [129]. Yet, the rapid advancements of LC–MS/MS in recent years provide a promising future for sHLA.

Taken together, the field of sHLA research has undergone a relatively slow development. Fifty years after the discovery of sHLA, fundamental questions still remain about these complex molecules and their underlying biology. Recent studies, however, suggest that we should renew our interest in sHLA to aid diagnosis and therapy design.

## Figures and Tables

**Table 1:** **Soluble HLA Class I Molecules in Malignancy**. Levels of soluble HLA class I molecules in various solid and hematological tumors. All studies measured sHLA levels by ELISA, unless indicated otherwise under Notes. * = mean ± SD, ** = mean ± SE, *** = median (range), **** = median [interquartile range].

Disease	N for sHLA Measurements	Treatment	Sample Type	Antibodies	sHLA Type	sHLA Levels Normal Controls	Correlation with Progression/Prognosis	sHLA Compared to Normal Controls	Notes
***Solid tumors***									
Gastric cancer (GC)	Healthy: 15, GC: 74	Unknown	Serum	W6/32 + IOT2	sHLA-I	868.9 ± 715.0 ng/mL *	Lower sHLA-I in higher stadia	↓	Lowest sHLA levels in stage IV. HLA-A24+ patients contained higher sHLA-I levels [14]
Pancreatic cancer (PC), hepatocellular carcinoma (HCC), gallbladder cancer (GBC), cholangiocellular carcinoma (CCC)	Healthy: 22, PC: 19, HCC: 51, GBC: 6, CCC: 6	Unknown	Serum	W6/32 + IOT2	sHLA-I	1470 ± 95.3 ng/mL **	N.A.	↑	[17]
Uveal melanoma	108	Yes (4), no (104). All undergoing enucleation	Aqueous humor	W6/32 + anti-β2M	sHLA-I	N.A.	Higher sHLA-I in higher stadia, bigger tumor prominence, ciliary body involvement, metastasis and poorer survival	↑	Magnetic Luminex; highest levels in stage III and IV [18]
Small-cell lung cancer (SCLC) and non-small-cell lung cancer (NSCLC)	Healthy: 84, SCLC: 23, NSCLC: 114	*n* = 6 for chemotherapy >3 months before blood collection	Plasma	W6/32 + anti-β2M	sHLA-I	sHLA-I: 1370 ng/mL (274–2670) ***	sHLA-I under cut off had significant prolonged OS	↑	[19]
Various malignancies	Randomly selected malignancies: 75; Infection that was cause of effusion: 21; Other pathologies: 21	Unknown	Body cavity fluids (70 pleural and 53 peritoneal)	W6/32	sHLA-I	Other diseases: 0.49 ± 0.45 ug/mL **	N.A.	N.A.	Competitive ELISA. Trend towards higher sHLA-I in malignant and infected patients compared to others, but not significantly different [13]
***Hematological tumors***									
Non-Hodgkin’s lymphoma (NHL) and Hodgkin’s disease (HD)	Healthy: ?, NHL: 65, HD: 37	Treatment naive	Serum	W6/32 + anti-β2M	sHLA-I	Unknown	NHL: higher sHLA-I levels are associated with shorter survival. HD: none found	↑	sHLA-I levels are higher pre-therapy [15]
NHL and HD	Healthy: 50, NHL: 40, HD: 9	Blood collection before initiation treatment	Serum	W6/32 + FG2/2 (anti-β2m)	sHLA-I	0.89 ± 0.08 ug/mL **	None found	↑	No statistical differences between patients in remission and normal controls [20]
Multiple myeloma (MM)	Healthy: 96; MM: 103	Chemotherapy naive: 44. ≥1 round of systemic anti-myeloma treatment ≥19 days ago: 59	Serum	W6/32 + anti-β2M	sHLA-I	261 (44–1010) µg/L ***	Higher sHLA-I in stage III compared to stage II. sHLA-I is an independent prognostic factor for OS	↑	[21]
MM and monoclonal gammopathy of undetermined significance (MGUS)	Healthy: 30, MGUS: 30, MM: 103	After collection. Conventional chemotherapy (53), high-dose therapy with autologous PBSC support (38), untreated (12)	Serum	W6/32 (sHLA-I) + anti-β2M	sHLA-I	1.04 ± 0.92 ug/mL *	Higher in MM stages II–III compared to stage I, above cut off predicted shorter OS	↑	sHLA-I higher in more advanced stages [22]
Waldenstrom macroglobulinemia (WM) and IgM-MGUS	Healthy: 41, IgM-MGUS: 63, WM: 42	After collection. WM patients (19/42) received alkylating agents (15) or fludarabine (4) ± rituximab	Serum	W6/32 (sHLA-I) + anti-β2M	sHLA-I	0.69 (0.4–0.8) ug/mL ****	Higher median sHLA-I in WM patients with adverse prognosis markers	↑	sHLA-I higher in WM [23]
Acute myeloid leukemia (AML)	Healthy: 12, AML: 20	Blood collection before initiation chemotherapy	Serum	W6/32 + TP25.99	sHLA-I (minus sHLA-G)	Range: 0.1–0.6 ug/mL	N.A.	↑	[24]
AML and advanced myelodysplastic syndrome (MDS)	Healthy: ?, AML: 209, MDS: 98	Treatment naive	Plasma	W6/32 + anti-β2M	sHLA-I	Unknown	AML: correlation with survival, complete response and cumulative response duration. MDS: none found	↑	Earlier transfusion or growth factor administration at earlier points could not be ruled out [16]

**Table 2:** **Soluble HLA-G Molecules in Malignancy**. Levels of soluble HLA-G molecules in various solid and hematological tumors. All studies measured sHLA levels by ELISA, unless indicated otherwise under Notes. * = mean ± SD, ** = mean ± SE, *** = median (range), **** = median (interquartile range), # = mean, $ = median ± SE, and ^ = mean (range).

Disease	N for sHLA Measurements	Treatment	Sample Type	Antibodies	sHLA Type	sHLA Levels Normal Controls	Correlation with Progression/Prognosis	sHLA Compared to Normal Controls	Notes
***Solid tumors***									
Papillary thyroid carcinoma (PTC)	Healthy: 80, PTC: 85	Unknown	Plasma	MEM-G/9 + anti-β2M	sHLA-G	3.51 (0–10.60) ng/mL ****	Decreased in patients with invasion. No difference before/after thyroidectomy.	↓	Tendency towards lower sHLA-G, not statistically different [41].
Thyroid cancer (TC)	Healthy: 45, TC: 40	Unknown	Serum	ELISA kit (Bio-Vendor)	sHLA-G	Unknown	N.A.	↓	Significantly lower serum HLA-G positivity [40].
Colorectal cancer (CRC)	Healthy: 113, CRC: 178	Treatment naive	Plasma	ELISA kit (Exbio)	sHLA-G1 and -G5	25.4 (3.6–97.1) U/mL ***	Higher in dead than alive patients. Above cut off associated with worse prognosis.	↑	[32].
Colorectal cancer (CRC)	Normal: 60, hyperplastic polyp: 72, IBD: 57, adenoma: 65, CRC: 144	Treatment naive	Serum	ELISA kit (Exbio)	sHLA-G1 and -G5	25.0 (13.8–39.5) U/mL ****	None found.	↑	Higher sHLA-G in all diseases, highest in CRC [35].
Colorectal cancer (CRC)	Mucinous carcinoma: 16, adenocarcinoma: 117	Unknown	Plasma	ELISA kit (Exbio)	sHLA-G1 and -G5	N.A.	Stage II: Negative correlation sHLA-G and liver metastasis free survival (LMFS). Stage III: Positive correlation sHLA-G and LMFS.	N.A.	[43].
Colorectal cancer (CRC)	Healthy: 10, CRC: 20	No prior treatment for CRC	Saliva and serum	ELISA kit (Bio-Vendor)	sHLA-G1 and –G5	Saliva: 6.3 U/mL, (3.4–10.4 U/mL) ^. Serum: undetermined	Higher saliva and serum sHLA-G levels in stage III–IV than stage I–II.	↑	Higher serum sHLA-G in age group >70 years [34].
Metastatic colorectal cancer (mCRC)	mCRC: 40	Untreated	Plasma	ELISA kit (Bio-Vendor)	sHLA-G1 and -G5	N.A.	Higher sHLA-G levels in patients with more than 1 metastatic site.	N.A.	[49]
Gastric cancer and colorectal cancer (CRC))	Healthy: 45, gastrointestinal cancer: 82	≈ 50% pre-operative chemo- or radiotherapy	Plasma	ELISA kit (Exbio)	sHLA-G1 and -G5	56.99 ± 48.45 U/mL **	Correlation between increased sHLA-G and stage I tumors.	↑	[50].
Gastric cancer (GC)	Healthy: 77, Benign disease: 53, GC: 81	No preoperative chemotherapy or radiotherapy	EDTA plasma	ELISA kit (Exbio)	sHLA-G1 and -G5	29.4 (24.0–39.3) U/mL ****	None found.	↑	Also higher sHLA-G levels in cancer compared to benign disease [36].
Hepatocellular carcinoma (HCC)	Healthy: 25, liver cirrhosis: 25, HCC: 36	Liver cirrhosis treatment naive, HCC unknown	Serum	ELISA kit (Bio-Vendor)	sHLA-G	47.0 ± 15.5 U/mL *	N.A.	↑	Also higher sHLA-G levels in cancer compared to liver cirrhosis [37].
Prostate cancer (PC)	Healthy: 26, Benign PC: 26, Malignant PC: 26	Unknown	Serum	ELISA kit (Elab-science Bio-technology)	sHLA-G	0.83 ng/mL #	A trend towards higher levels in more advanced stage.	↑	A statistical difference observed between the three groups, but no apparent difference between benign/malignant PC [38].
Endometrial cancer (EC)	Healthy: 45, EC: 40	Treatment naive	Plasma	ELISA kit (Exbio)	sHLA-G1 and -G5	0.145 ± 0.102 ng/mL **	Higher sHLA-G in high grade disease than low grade (trend).	↑	Indicated as trend, as borderline significant (*p* = 0.057) [45].
Ovarian pathologies (including ovarian cancer)	Benign serous ovarian cysts (G1): 54, Endometriosis (G2): 43, Ovarian cancer (G3): 38	Before chemotherapy: 31, after: 7 (ovarian cancer patients)	Perito-neal fluid (PF) and serum	ELISA kit (Bio-Vendor)	sHLA-G	N.A.	PF sHLA-G concentrations and the difference between sHLA-G in serum and PF can differentiate G2 and G3.	N.A.	No significant differences in both PF and serum sHLA-G levels between groups. The median difference between PF and serum sHLA-G levels significantly higher in ovarian cancer patients [39].
(Neoadjuvant-treated) breast cancer (BC)	Healthy: 16, BC: 190	All undergoing neoadjuvant chemotherapy	EDTA plasma	MEM-G/9 and anti-β2M	sHLA-G	16.3 (4.0–37.8) ng/mL ***	Before therapy: sHLA-Gev above cut off associated with worse PFS, sHLA-Gfree above cut off with better PFS and OS.	↑	[51].
Breast cancer (BC)	Healthy: 84, BC: 75	Most patients received chemo-, radio- or hormone therapy	Plasma	ELISA kit (Qayee Biological Technology Co., Ltd.)	sHLA-G	Median: 22.71 ng/mL. Mean: 31.58 ± 28.60 ng/mL	sHLA-G levels could identify mastectomized patients.	↓	61 patients were mastectomized [44].
Head and neck squamous cell carcinoma (HNSCC)	Healthy: 99, HNSCC: 120	Treatment naive	Serum	SPR: Mem-G/9, ELISA: kit (Cloud Clone Corp.)	sHLA-G	ELISA: 2.47 ± 0.10 ng/mL *	Seemingly higher sHLA-G in advanced stages.	↑	Surface plasmon resonance (SPR) and ELISA [33].
Neuroblastoma (NB)	Healthy: 13, NB: 31	Unknown	Bone marrow plasma	ELISA kit (Exbio)	sHLA-G	25.16 ± 7.38 ng/mL $	sHLA-G higher in stage 3–4 than stage 1–2.	__	[46].
Melanoma	Healthy: 126, Melanoma: 190	Cytostatics (24), IFN-α (31), or treatment naive (135)	Serum	ELISA: W6/32 + anti-β2M	sHLA-G	22.92 ± 1.51 ng/mL **	None found.	↑	Prior to ELISA serum depletion with TP25.99. IFN-α treatment was the only factor of impact on increased sHLA-G levels [52].
***Hematological tumors***									
Acute lymphoblastic leukemia (ALL)	Healthy: 14, ALL: 33	Before/after intensification phase of chemo-therapy (IPC)	Serum	ELISA kit (Biovendor)	sHLA-G	31.0 ± 21.1 U/mL *	None found.	↑	Before IPC non-significantly increased, after IPC significantly increased [47].
T cell acute lymphoblastic leukemia (T-ALL)	T-ALL: 32	Chemotherapy	Bone marrow (BM) aspirates	ELISA kit (Biovendor)	sHLA-G	N.A.	Association with leukocytosis at diagnosis. Not related to survival.	N.A.	Patients’ age: 2–16 years. sHLA-G levels did not change during treatment [53].
Acute myeloid leukemia (AML)	Healthy: 15, AML: 30	Unknown for newly diagnosed patients, conventional chemotherapy for relapsed patients	Serum	ELISA kit (Glory Science)	sHLA-G1 and -G5	329.8 ± 57.54 ng/mL *	Higher in relapsed patients than newly diagnosed. patients and related to bone marrow blasts percentage.	↑	Newly diagnosed: 15, Relapsed: 15 [54].
Chronic myeloid leukemia (CML)	CML: 68	Imatinib, nilotinib or dasatinib	EDTA plasma	ELISA kit (Bio-Vendor)	sHLA-G1 and -G5	N.A.	High secretor haplotype is associated with lower event-free survival during TKI treatment.	N.A.	Higher sHLA-G in high secretor haplotype (HLA-G*01:01:01/02) than low secretor haplotyope (HLA-G*01:01:03) [55].
Waldenstrom macroglobulinemia (WM) and IgM-MGUS	Healthy: 41, IgM-MGUS: 63, WM: 42	After serum collection. WM patients (19/42) received alkylating agents (15) or fludarabine (4) ± rituximab	Serum	MEM-G/9 (sHLA-G) + anti-β2M	sHLA-G	17.3 (12–21) ng/mL ****	None found.	↑	sHLA-G higher in WM and IgM-MGUS [23].

**Table 3:** **Soluble HLA-E and HLA-F Molecules in Malignancy**. Levels of soluble HLA-E and -F molecules in various solid and hematological tumors. All studies measured sHLA levels by ELISA, unless indicated otherwise under Notes. * = mean ± SD, *** = median (range), # = mean, $ = median ± SE, and ^ = mean (range).

Disease	N for sHLA Measurements	Treatment	Sample Type	Antibodies	sHLA Type	sHLA Levels Normal Controls	Correlation with Progression/Prognosis	sHLA Levels Compared to Normal Controls	Notes
***Solid tumors***									
Melanoma	Healthy: 94, Melanoma: 127	No current therapy	Serum	MEM-E/08 + MEM-E/07	sHLA-E	0 (0–1224) pg/mL ***	None found.	↑	Mostly higher in stage III [56].
Neuroblastoma (NB)	Healthy: 75, NB: 84	Unknown	Plasma	sHLA-E: 3D12 + anti-β2M, sHLA-F: polyclonal anti-HLA-F + anti-β2M	sHLA-E and sHLA-F	sHLA-E: 0.07 ± 0.02 units/mL * and sHLA-F: 0.1 ± 0.03 units/mL *	Higher sHLA-E and -F levels (above cut off) associated with better EFS and OS.	↑	[48].
Neuroblastoma (NB)	Healthy: 13, NB: 31	Unknown	Bone marrow plasma	3D12 + anti-β2M (Exbio)	sHLA-E	48.01 ± 10.93 U/mL $	sHLA-E higher in stage 3–4 than stage 1–2.	↓	Tendency towards lower sHLA-E, not statistically different [46].
Nasopharyngeal carcinoma (NPC)	Healthy: 65, NPC: 81	Unknown	Plasma	Commercial kit (TSZ)	sHLA-F	10.06 (3.22–74.05) pg/mL ^	N.A.	↑	Tendency towards higher sHLA-F, not statistically different [57].
***Hematological tumors***									
Chronic lymphocytic leukemia (CLL)	Healthy: 35, CLL: 50	Chemotherapy naive	Plasma	3D12 + MEM/07	sHLA-E	1222 ± 101 pg/mL *	HLA-E*01:03 is an independent predictor for disease progression.	___	No difference between healthy and CLL, but significantly higher in stage C than stage A. sHLA decreases after therapy [58].
Acute leukemia (AL)	Healthy: 64, AL: 54	Unknown	Plasma	Commercial kit (Shang-hai Jianglai Biotech. Co. Ltd.)	sHLA-E	18.34 pg/mL #	N.A.	↑	[59].

**Table 4:** **Soluble HLA Class II Molecules in Malignancy**. Levels of soluble HLA class II molecules in solid and hematological tumors. All studies measured sHLA levels by ELISA, unless indicated otherwise under Notes. ** = mean ± SE.

Disease	N for sHLA Measurements	Treatment	Sample Type	Antibodies	sHLA Type	sHLA Levels Normal Controls	Correlation with Progression/Prognosis	sHLA Levels Compared to Normal Controls	Notes
***Solid tumors***									
Malignant melanoma	Healthy (random): 86, Healthy (old): 35, Melanoma: 183	Cytostatic drugs, immunomodulatory agents or nothing.	Serum	1.243 + CR3/43	sHLA-DR	Healthy (random): 1.38 ± 0.16 ug/mL; Healthy (old): 1.519 ± 0.1 ug/mL **	Independent predictor of progression-free survival.	↓	[60].
***Hematological tumors***									
Pre-B acute lymphoblastic leukemia (ALL)	Healthy: 31, ALL; 30	Treatment naive	Plasma	Polyclonal rabbit HLA-DRB1 + mAb anti-HLA-DR clone L243	sHLA-DRB1	0.051 ± 0.007 ug/mL **	N.A.	↑	[61].
